# Supplementary material for: Are carbon emissions trading and green financial instruments synergistic? -Comprehensive quantitative research based on content analysis
Source: PLoS One. 2024 Mar 7;19(3):e0298601. doi: 10.1371/journal.pone.0298601 (PMC10919601; doi:10.1371/journal.pone.0298601)
Supplement: S1 Data — (ZIP) [file pone.0298601.s001.zip › Data/地级市Z_F工作报告.zip!/╣π╬≈/┴°╓▌╩╨/└┤╫╘╣1⁄2╓┌║┼í╛┬φ┐╦╩2╛▌═°í┐_2016.docx]

各位代表：

现在，我代表市人民政府向大会作政府工作报告，请予审查，并请各位政协委员和列席会议的同志提出意见。

一、2015年和“十二五”工作回顾

过去的一年，面对复杂多变的形势和艰巨繁重的改革发展稳定任务，我们坚决贯彻落实习近平总书记系列重要讲话精神，认真落实中央 、自治区 、市委关于稳增长 、促改革 、调结构 、惠民生 、防风险的各项决策部署，努力化解经济下行压力，积极应对挑战，保持了经济社会持续健康发展，经济运行总体平稳 、稳中有进 、稳中有好。预计全市实现地区生产总值2311亿元，增长8.0%；工业总产值4573亿元，增长4.0%；固定资产投资2048亿元，增长16.0%；财政收入343.8亿元，增长8.6%；社会消费品零售总额940亿元，增长9.5%；城镇居民人均可支配收入29362元，增长10.0%；农民人均纯收入9553元，增长11.0%。城镇登记失业率3.95%，居民消费价格指数全年累计上涨1.7%。

经济基本面保持稳定。工业结构调整取得实效，财政支持企业发展专项资金达6.5亿元，完成更新改造投资1058亿元，增长15%。汽车工业产值2082.6亿元 、产量229.1万辆，城市汽车产量占全国比重达9.7%，排名由全国第5位上升到第3位；冶金 、机械工业积极调整产品结构，战略性新兴产业规模以上工业总产值突破400亿元，亿元以上工业企业达429家。柳东新区成为广西首个产值超千亿元开发区，河西工业园区成为广西第八个自治区级高新技术产业开发区，柳江县新兴工业园列为自治区产城融合发展试点园区，鹿寨经济开发区成为广西工业园区“两化”融合试点，园区工业集中度达61%。农业现代化步伐加快，柳江荷塘月色 、鹿寨呦呦鹿鸣 、三江布央茶产业等现代特色农业（核心）示范区通过自治区级验收，落久水利枢纽工程动工建设，实施7万亩“双高”糖料蔗基地建设。“倍增计划十大产业工程”完成创建特色种植业基地54万亩 、农业观光旅游村（园）35个 、万元示范村100个 、十万元示范农户500户。特色农产品品牌推广力度加大，融 安金桔入选2015中国果品区域公用品牌50强。养殖业加快规模化 、集约化 、标准化生产。主要农作物耕种收综合机械化水平达54%。“美丽柳州·生态乡村”建设扎实推进，53个市级“美丽柳州”乡村建设综合示范村屯治理成效明显，303个自治区级绿化示范村屯建设扎实推进，乡村环境明显改观。现代服务业提速发展，服务业实现增加值828.6亿元，增长12.0%，增速比上年提高2.5个百分点。万达广场 、窑埠古镇 、风情港等大型商业综合体建成开业，新柳邕农产品批发市场（一期）投入运营，鹧鸪江钢铁深加工及物流产业园被评为全区示范物流园区。

“电商柳州”稳步实施，新增电商企业266家，全市电子商务交易额444.9亿元，增长31.5%； 柳州螺蛳粉借助电商平台发展成为新兴产业，成为广西最热电商产品和广西最具影响力十大特产之一；柳城县被评为国家电子商务进农村示范县。金融业发展稳定，新增各类金融机构5家，小额贷款公司实现县域全覆盖，设立了广西首家城商行小微企业金融服务中心；在全区率先成立市现代农业融资性担保有限公司，搭建农业产业化 、农村综合产权交易和新农村建设投融资担保平台；银行业本外币存 、贷款余额分别为2822.7亿元和2051.4亿元，均位居全区第二。旅游名城建设深入推进，融水县 、三江县积极创建广西特色旅游名县，凤凰河生态旅游度假区成为国家4A级旅游景区，全市旅游总收入280亿元，增长20%。

城市综合功能不断完善。柳北沙塘、鹿寨寨沙列入全国建制镇示范试点。湘桂铁路柳州至南宁段电气化改造完成，柳州站站房扩建工程、柳州东站还建等项目开工建设，柳州至武宣高速公路建成通车，白莲机场航站楼及配套设施扩建、柳州至石龙三江口Ⅱ级航道进展顺利，广雅大桥、柳铁中心医院1号住院楼荣获国内建筑行业工程质量最高荣誉奖—— —“鲁班奖”。打击“两违”的“风暴行动”和市容环境综合整治“百日大会战”有效开展。“生态花园·五彩画廊”工程进展顺利，城市环境空气质量预报预警系统建成使用，市区空气二氧化硫、二氧化氮年均值稳定在国家二级标准，可吸入颗粒物（PM10）、细颗粒物（PM2.5） 均值分别下降23.9%和25.4%，柳江河饮用水保护河段继续保持国家地表水Ⅲ类以上水质标准。全面完成自治区下达的“十二五”节能减排目标任务。

民生保障力度加大。为民办十件实事全面完成。市本级财政用于民生支出102.1亿元，占一般公共预算支出的77.3%。推进精准扶贫，落实财政专项扶贫资金2.2亿元，7000多名干部进驻村屯，精准识别到村到户到人，全年实现5万贫困人口脱贫。保障性安居工程实物建房开工15193套、基本建成12014套、分配入住23832套。农村危房改造开工21397户，竣工12414户。

投资400万元为北部三县25.6万户农房购买保险，受损农房获得理赔金额484.3万元。市区城市低保标准由每人每月390元提至480元，市区农村低保标准由每人每年2400元提至3000元。

居家养老和社区养老覆盖全部城市社区和30%的农村社区，为8万多名高龄老人发放高龄补贴。

各项社会事业全面发展。“文化惠民”成果丰硕，新排精品大剧《侗寨人家》成功展演，新建84个村级公共服务中心，188个行政村实现有线电视联网。教育资源进一步优化，创建自治区多元普惠幼儿园108所，开展市区学校义务教育段学区制办学改革探索，推进名校教育集团化，取消公办普通高中招收择校生政策和“三限”政策。成功创建国家卫生城市，大力推行分级诊疗机制，全面实行药品零差率销售，异地就医广西区内实现结算“一卡通”。诚信计生实现全覆盖，出生人口性别比控制在合理区间。

“平安柳州”建设深入开展，迅速侦破柳城“9·30”爆炸案，稳妥做好善后工作。深入创建全国文明城市，积极培育和践行社会主义核心价值观。民族团结进步事业不断发展。积极开展“双拥”共建，支持国防和军队建设，开展纪念中国人民抗日战争暨世界反法西斯战争胜利70周年系列活动，国防教育、优抚安置等工作持续加强，军政军民关系进一步密切。

政府自身建设不断加强。扎实开展“三严三实”专题教育，政风行风进一步好转。做好“简政放权、放管结合”，优化行政审批体制，实施“简政惠民、优化服务”为主题的“八个一”综合改革试点，在全区率先成立市行政审批局，由一枚行政审批专用章取代35个部门、单位原82枚行政审批章。坚持依法行政，建设法治政府，出台《柳州市推行政府部门权力清单的实施意见》，成立政府法律顾问室，执行行政机关负责人出庭应诉制度。法治柳州建设卓有成效，法制宣传深入开展，“六五”普法圆满完成。严格执行市人大及其常委会的各项决议决定，自觉接受市人大及其常委会的法律监督和工作监督、市政 协的民主监督，办理市人大代表议案3件、建议131件，办结率100%，满意率98.4%；市政协委员提案183件，办结率100%，满意率100%。

五年来，全市经济社会发展不断取得了新成绩，实现了“十二五”的顺利收官。

（一）经济综合实力迈上新台阶。

“十二五”期，全市地区生产总值年均增长9.8%，工业总产值年均增长11.7%，固定资产投资年均增长21.8%，财政收入年均增长11.3%，社会消费品零售总额年均增长14.4%。工业发展继续领跑全区，经济总量大幅提升，在广西发展战略中占有重要地位。

（二）产业转型升级进入新阶段。

三次产业结构进一步优化，服务业增加值年均增长10.4%。战略性新兴产业占全市比重由2010年的2.1%提升到了10%；更新改造投资累计完成4057.6亿元，是“十一五”时期的4倍。工业不断做大做强做优，服务业比重持续提升，农业基础进一步夯实，城乡经济社会协调发展。

（三）城乡建设展现新面貌。柳州被列为国家新型城镇化综合试点，城镇化率由2010年的55.1%提升到62%左右，市区建成区面积由135.06平方公里扩增至183.92平方公里。

“绿满龙城”工程、“生态花园·五彩画廊”花园城市建设成效明显，森林覆盖率由2010年的63.2%增长为64.9%，城市建成区绿地覆盖率由40.4%提高到43.2%，人均公共绿地由10.1平方米增加至12平方米。

“十二五”时期农田水利、农村公路建设累计完成投资10.4亿元和25.1亿元，分别是“十一五”时期的3.6倍和2倍，解决了89.7万农村人口饮水安全问题；建设农村公路2793公里，新增通沥青（水泥）路行政村277个。

（四）全面深化改革取得新进展。

深化国资国企改革，国有资产实现保值增值，市属国有及国有控股企业资产总量是2010年的5.6倍，净资产是2010年的6.7倍。新型城镇化综合改革试点扎实开展，建立PPP项目储备库并逐步向社会发布。推进农村集体产权制度改革，开展农村土地承包经营权确权工作，农户家庭承包土地经营权流转面积50万亩。全面推行注册资本认缴登记制度和“三证合一”等工商登记制度改革，全市新设立企业14951户，增长111%。金融领域改革不断深化，“十二五”期末银行业本外币存、贷款余额分别是“十一五”期末的1.89倍和1.99倍，全市金融机构达82家、新型金融组织65家、上市企业16家，提前两年完成自治区下达的企业上市目标任务。文化、教育、卫生、财税等领域改革统筹推进，经济社会发展释放新活力。

（五）开放合作实现新拓展。“十二五”时期共引进市外境内到位资金2832亿元，实际利用外资18亿美元，分别是“十一五”时期的2.2倍和2.6倍。积极融入国家“一带一路”和珠江-西江经济带发展战略，柳州保税仓申报成功，上汽通用五菱、柳工等企业加快在国内外设立生产基地。连续成功举办五届中国-东盟（柳州）汽车、工程机械及零部件博览会。国家汽车及零部件出口基地出口增幅全国排名第一。全市对外投资位居广西首位。

（六）人民生活水平获得新提升。

城镇居民人均可支配收入和农民人均纯收入年均增长分别为10.6%和14.1%。“十二五”期累计新增城镇就业29.2万人、新增农村劳动力转移就 业29.3万人。“文化建设十大工程”加快推进，柳州工业博物馆成为文化新地标，成功打造“中外优秀剧目月月演”等文化惠民平台，文艺佳作不断涌现，国际水上狂欢节、国际奇石节、世界水上极速运动大赛成为国内外有影响力的特色文化品牌。市区义务教育均衡发展学校基本建设三年攻坚规划新建、改建学校24所，已竣工12所，规划62个扩建项目已竣工47个，新增班级859个，全面推进农村义务教育薄弱学校改造，学前教育三年毛入园率76%、义务教育巩固率97%、高中阶段教育毛入学率92%。

改制企业职工危旧房集中区改造工程累计投入105亿元，惠及97家改制企业和1.3万户职工家庭，棚户区改造任务超额完成。累计完成8.57万户农村危房改造。连续十一年上调城镇职工基本养老金。民生和社会事业持续改善，社会大局保持和谐稳定。

过去五年，全市人事、编制、外事、侨务、口岸、工商、税务、质量技术监督、食品药品监管、统计、审计、国家安全、决策咨询、档案、地方志、气象、粮食、供销、宗教、老龄、妇女儿童、防汛抗旱、防震减灾、社会科学等各项工作都取得了新的成绩，作出了新的贡献。

各位代表，五年的成绩来之不易。

这是党中央、国务院和自治区党委、自治区人民政府以及市委正确领导的结果，是历届党委、人大、政府、政协打下的良好基础和全市各族人民精诚团结、艰苦奋斗的结果。在此，我代表市人民政府，向全市人民，向驻柳人民解放军、武警部队官兵，向各民主党派、工商联、人民团体及海内外所有关心和支持柳州发展的各界人士，致以崇高的敬意和衷心的感谢！在总结成绩的同时，我们也清醒地认识到，受宏观环境、发展阶段、经济结构等多重因素影响，柳州经济社会发展不平衡、不协调、不可持续问题仍较为突出，主要是：经济运行不稳定因素增多，稳增长压力很大；产业转型升级任重道远，战略性新兴产业比重较低，新的增长点尚未形成；自主创新能力有待提升，创新驱动发展的基础还不牢固；环境治理任务较重，大气和水污染问题时有发生；公共服务水平仍需不断提高，脱贫攻坚任务还很艰巨；转变政府职能、优化发展环境任务依然繁重。

对此，我们将不断改进和提升政府工作水平，采取更有针对性的举措认真加以解决。

二、“十三五”时期的奋斗目标和主要任务

“十三五”时期，是柳州在全区率先全面建成小康社会的决战期，是打造西江经济带龙头城市的关键期，更是奋力实现“经济升级，城市转型”新跨越的攻坚期。这五年的发展，对柳州的未来至关重要。刚刚结束的中共柳州市第十一届代表大会第三次会议，为柳州新发展描绘了美好蓝图。我们要认真贯彻中共柳州市第十一届代表大会第三次会议精神，准确把握柳州所处的历史方位和阶段性特征，准确把握新常态下的新趋势新特点，奋力走出一条具有柳州特色的发展道路。

“十三五”时期柳州发展的指导思想和战略思路是：全面贯彻党的十八大和十八届三中、四中、五中全会精神，深入贯彻习近平总书记系列重要讲话精 神，按照“五位一体”总体布局和“四个全面”战略布局，围绕自治区党委十届六次全会部署和“两个建成”奋斗目标，坚持发展第一要务，以提高发展质量和效益为中心，牢固树立创新发展、协调发展、绿色发展、开放发展、共享发展理念，深入实施“实业兴市，开放强柳”战略，充分发挥实业引擎和开放引擎作用，加快形成引领经济发展新常态的体制机制和发展方式，全面加快结构调整和转型升级，进一步做强产业、做好民生、做优环境、做大城市，力争在全区率先全面建成小康社会，建设广西构筑“一带一路”有机衔接门户的重要节点，打造成为产业集聚、要素集聚、人口集聚的西江经济带龙头城市。

根据《中共柳州市委员会关于制定柳州市国民经济和社会发展第十三个五年规划的建议》，市人民政府在广泛征求党代表、人大代表和政协委员、各县区各部门、专家学者以及社会各界意见的基础上，制定了《柳州市国民经济和社会发展第十三个五年规划纲要（草案）》，整个起草过程充分体现了科学决策和民主决策的要求，是集体智慧的结晶。

《规划纲要（草案）》围绕主题、突出主线，提出经济发展、民生福祉、创新驱动、生态文明四大类31项主要目标，其中约束性15项、预期性16项。明确了“十三五”期全市地区生产总值年均增长8％，经济增速高于全国、全区水平，提前实现地区生产总值比2010年翻一番；实现规模以上工业增加值年均增长8%，财政收入年均增长8％，固定资产投资年均增长12%，社会消费品零售总额年均增长10％；城乡居民收入保持两位数增长，提前实现城乡居民收入比2010年翻一番的目标。这些目标体现质量、效益、结构、速度的统一，体现经济发展与改善民生的统一，体现先行先试与可行可为的统一，经过全市人民共同努力是可以实现的。

《规划纲要（草案）》分7篇27个章节，提出了今后五年全市经济社会发展的主要任务，归纳起来有以下几个方面：（一）实施创新驱动战略 ，全面推进实业兴市。着力增强企业自主创新能力，打造公共创新平台，构建金融科技融合体系，积极创建国家创新型试点城市和广西自主创新示范区。着力优化制造业产业布局，构建“一圈两核三片”工业产业布局，努力把柳州建成国家重要的先进制造业基地。着力打造现代特色农业“二圈七带四区”总体布局，促进农业产业化发展，推进现代特色农业（核心）示范区建设，不断拓展农民增收渠道；着力提升发展现代服务业，实施服务业发展十大工程，打造区域性产业金融服务中心，形成产业联系紧密、上下游协调、结构合理的现代服务业体系，到2020年服务业增加值占地区生产总值比重达到40％。

（二）实施开放发展战略 ，全面推进开放强柳。以拓展对外交通新格局、加快区域产业集聚发展、扩大对外开放合作为重点，进一步扩展区域发展空间，不断构筑对外开放合作新优势。主要任务是积极融入国家“一带一路”战略，建设成为全国重要的综合交通枢纽；积极参与珠江-西江经济带开放开发，构建全方位对外开放新格局；坚持“引进来”与“走出去”，全面提升开放型经济水平。

（三）实施协调发展战略 ，全面推进新型城镇化。实施新型城镇化建设行 动计划，打造“一圈一带四轴”城镇空间组织结构和柳来、柳桂经济发展带城镇群。深入推进“美丽柳州”乡村建设，完善农村基础设施与社会服务体系。进一步提升城市综合服务功能，加快打造现代都市经济圈。到2020年，常住人口城镇化率达到68.7％，把柳州建设成为市区建成区面积200平方公里以上、常住人口200万人以上、引领西江经济带发展的龙头城市。

（四）实施绿色发展战略 ，打造美丽柳州新形象。加快形成绿色发展方式和生活方式，努力创建国家生态文明先行示范区。实施“生态柳州”建设工程，加强环境保护和生态建设，创建国家环境保护模范城市；大力推进清洁生产工作，积极发展循环经济，创建国家循环经济示范城市；推动绿色发展，改善生态环境，积极创建国家生态市、国家生态园林城市。

（五）实施共享发展战略 ，提升幸福柳州新水平。重点实施脱贫攻坚“十大行动”，打赢精准扶贫攻坚战，2019年前确保现行标准下的农村贫困人口实现脱贫销号，融水、三江、融安三个贫困县和313个贫困村全部摘帽出列。实施城乡居民收入倍增计划和提高社会保障行动计划，增强文化、教育、医疗卫生等公共服务能力，让人民群众在共建共享中拥有更多获得感。

（六）全面强化规划保障 ，奋力实现“十三五”蓝图。全面推进深化改革，重点推进行政体制、国资国企、农村产权制度、户籍制度以及市场体系、新型城镇化投融资机制、财税体制等领域改革，加快构建现代金融服务体系，鼓励、支持、引导非公有制经济加快发展。全面推进“法治柳州”建设，完善立法体制机制，加快法治政府建设，健全社会信用体系。通过明确政府职责，科学引导社会资源，动员全社会力量保障纲要顺利实施。

各位代表，柳州“十三五”发展的宏伟蓝图已经绘就，目标已经明确，关键在于落实。我们将在市委的正确领导下，与全市各族人民一道，同舟共济、万众一心，一步一个脚印把规划落实到具体项目、具体工作上，不断克服前进中的困难，不断增创发展中的新优势。

我们坚信，经过全市人民的团结奋斗，柳州的明天一定会更加美好！

三、2016年的主要目标与重点工作

2016年是“十三五”的开局之年，也是推进结构性改革的攻坚之年。做好今年的政府工作，意义重大。

今年柳州经济社会发展的主要预期目标是：地区生产总值增长7.6%以上，规模以上工业增加值增长6.5%，固定资产投资增长14%，财政收入增长7%，社会消费品零售总额增长10%，城镇居民人均可支配收入增长10%，农民人均纯收入增长11%。城镇登记失业率和单位生产总值能耗、主要污染物排放总量控制在自治区下达指标以内。

对于以上预期目标，主要出于以下几方面因素考虑：一是充分考虑到发展面临的形势和压力。当前，经济下行压力持续加大，截至去年12月全国工业品出厂价格（PPI已经连续46个月下降；到去年12月中国制造业采购经理指数（PMI为49.7%，连续5个月低于50%，说明制造业处于收缩空间，更深层次地反映出传统行业、高耗能行业仍在深度调整之中。去年柳州钢铁、机械、化工三大行业产值比上年减少235亿元，拉低全市工业产值增速5.5个百分点。今年这些行业下行趋势仍比较明显，所以我们面临的挑战将更为严峻，稳增长任务艰巨而繁重。二是充分考虑了市委“十三五”规划建议提出经济增速高于全国、全区水平的要求，考虑了柳州要在全区率先全面建成小康社会，提前实现地区生产总值、城乡居民人均收入比2010年翻一番的要求。三是充分结合柳州自身实际。制定“地区生产总值增长7.6%以上”目标，主要是与自治区增长7.5%~8%的预期目标保持一致，体现柳州必须为广西经济发展多作贡献；制定“规模以上工业增加值增长6.5%”的目标，主要是考虑企业生产经营面临的困难增多，我们要支持企业提高生产经营的质量和效益，给企业转型发展留足空间；制定“固定资产投资增长14%”的目标，就是要坚持把投资作为拉动经济增长最直接、最关键的手段，抓住自治区赋予柳州实施新型城镇化综合改革先行先试的机遇，加强基础设施建设，推进城镇化开发，加快城市化进程，通过投资拉动经济增长；制定“财政收入增长7%”的目标，主要是考虑进一步提高财政的质量，同时保证全市的民生保障支出与其他刚性支出。在实际工作中，我们将全面贯彻落实“创新、协调、绿色、开放、共享”的发展理念，真抓实干，力争实现更好的结果，努力为“十三五”开好局、起好步。今年，我们将重点抓好以下工作：（一）大力开展实体经济发展年活动 ，提高工业发展质量和效益加快产业优化升级。启动新一轮技术改造，更新改造投资增长10%以上。汽车产业要力争上汽通用五菱宝骏二期整车产能、东风柳汽乘用车发动机等项目建成投产，提升汽车零部件系统化、模块化、专业化水平。钢铁产业要加快柳钢冷轧汽车用钢开发，推动形成高附加值钢铁产品产业化。机械产业要推进智能化、集成化、机电仪一体化改造，提升新产品和核心零部件研发制造能力，开工建设柳工智能化工厂升级改造等项目。用高新技术和先进实用技术改造升级食品、化工、纺织服装、建材等传统产业。进一步促进企业做强做优，财政安排支持企业发展资金比上年增长20%以上。设立产业发展基金，扶持“专、精、特、新”中小微企业发展，力争亿元以上工业企业达440家。

坚持创新驱动发展。促进互联网与工业融合创新，编制《柳州智能制造2025》发展规划，支持东风柳汽数字化生产制造流程管理集成平台、柳钢集团制造执行系统（MES）等项目建设，实施上汽通用五菱新型SUV研发等技术创新项目。深入创建全国质量强市示范城市、国家级创新型试点城市，争创自治区级自主创新示范区，力争新增10家国家级高新科技企业、2家自治区级工程技术研究中心，引进1家国家级专业研究机构。

培育发展战略性新兴产业。主动对接“中国制造2025”“互联网+”产业布局，构建战略性新兴产业联盟。通过产业链垂直整合和创新资源优化组合，大力发展新能源汽车、电子信息、高端装备、新材料等产业，加快上汽通用五菱新能源汽车、东风柳汽混合动力汽车研发生产，实施一批市场前景好、自主创新能力强的项目，力争战略性新兴产业占全市规模以上工业产值比重超过11%。

抓好产业园区建设。着力打造特色产业园区，支持柳东新区申报成为国家产城融合示范区，加快电子信息产业园、中欧合作产业园、柳北循环经济产业园等园区建设。支持县区工业集中区发展，完善柳江县河表工业片区、柳城县工业区沙埔片区、融安县广西香杉生态工业园、柳北沙塘工业园（二期）等基础设施，推进洛维中小企业创业基地、柳南科技型中小企业孵化园、柳江县小微企业创业基地、鹿寨县中小科技企业创业孵化基地等建设，不断增强园区承载能力。

（二）大力开展重大项目建设年活动，扩大投资增强发展后劲狠抓重点项目建设。实施市级层面重大项目458项，年度计划投资666亿元，其中：新开工红花枢纽二线船闸工程 、凤凰岭大桥、柳化整体搬迁及技术改造等重大项目177个，竣工古偿河水库工程、环江滨水大道工程、西电东送500千伏桂中换流站工程等重大项目84个。加快桂林至柳州高速公路改扩建（柳州段） 、柳东综合交通枢纽 、空港经济区等111个重大项目前期工作，筛选一批项目列入国家 、自治区重点项目盘子，形成“开工一批 、竣工一批 、储备一批”的良好态势。

做好项目建设要素保障。创新融资方式，引入社会资本参与各类项目建设，鼓励社会资本进入教育 、医疗 、养老服务等社会事业领域以及城乡供水 、污水处理 、水利河道疏浚等基础设施领域。优先保障重点项目建设用地，最大限度挖掘土地潜力，提高土地利用效率。进一步落实项目建设主体责任，细化目标任务，强化考评督查机制，形成项目推进的强大合力。

加大招商引资力度。推动一批国内外知名企业入柳，补强原有产业，培育发展新产业。开展汽车产业 、生物医药 、电子信息 、智能制造 、新材料新能源等领域专题招商引资工作，重点抓好柳州电子信息产业园等招商项目落地建设。坚持以存量引增量，积极承接产业转移，优化外资结构，引进市外境内资金增长7%以上，利用外资（全口径）增长5%以上。

（三）大力开展精准脱贫推进年活动，保质保量完成全年脱贫任务全面完成精准识别建档立卡。用好精准识别成果，对贫困村贫困户逐村逐户建档立卡，做到一村一册 、一户一卡，构建覆盖市 、县 、乡 、村 、屯五级扶贫信息网络系统，全面掌握贫困村贫困户基本情况和帮扶成效。

开展精准脱贫攻坚。实施“七个一批”精准帮扶措施，确保完成年度脱贫8.5万人任务。启动“基础设施建设大会战”，集中解决贫困地区路 、水 、电 、房 、网络等瓶颈问题，年内完成通村道路全部硬化 、屯级道路硬化300公里 、农村危房改造1万户以上任务。发展特色富民产业，整合各类涉农资金，将50%以上资金投入到贫困地区，因地制宜推进特色优势产业发展。大力推进扶贫移民搬迁，制定好实施方案，有序推进分类搬迁，年内完成农村贫困人口搬迁7300人。

推进金融扶贫工作，支持扶贫龙头企业和贫困村农民专业合作社发展，年内金融扶贫贷款额度达2.6亿元以上，比2015年翻一番，着力解决贫困村贫困户发展生产资金短缺问题。

提高社会扶贫实效。引导企业（商会）开展“村企共建”“百家民营企业扶助百个贫困村”活动。开展“雨露计划”教育扶贫培训，落实适龄职教学生“一免一补一统筹”帮扶政策，确保每位建档立卡贫困家庭上学子女得到政策资助。

（四）提升农业现代化水平，统筹城乡区域协调发展优化农业产业结构。创建一批现代农业示范区，力争新增3个以上现代特色农业（核心）示范区通过自治区级验收。确保粮食生产稳定，粮食种植面积保持在248万亩以上。优化调整农业产业结构，发展水果 、桑蚕 、茶叶 、蔬菜 、水产畜牧养殖等特色农业，建设11万亩“双高”糖料蔗基地，推动农业规模化 、专业化 、标准化 、品牌化发展，促进农业提质增效。

实施“互联网+”现代农业行动，推进京东 、农村淘宝等农村电商项目建设。

推广运用农机生产，主要农作物耕种收综合机械化水平达57%以上。加强农产品质量安全监管，促进农业形成绿色安全生产。

加快城乡一体化进程。完善城乡交通路网，力争建成三江至柳州高速公路，实施罗城至洞头（融水段） 、国道321良口至梅林等国省道路网项目。

抓紧推进柳北沙塘 、鹿寨寨沙等国家建制镇试点，建设柳北片区新型城镇化项目，促进柳江 、鹿寨与市区同城化发展，统筹柳江规划建设，进一步拉开城市框架。推进北部四县新型城镇化建设，规划三柳高速生态经济带。

实施鹿寨江口及导江区域 、太阳村镇 、石碑坪镇 、沙塘镇等控规编制，着力构建科学合理的新型城镇化体系。

完善农村基础设施。做好落久水利枢纽工程移民安置和枢纽主体工程建设，加快推进洋溪水利枢纽工程前期工作。以土地流转为基础，实施“小块并大块”推动农村土地整治，重点抓好高标准农田建设，实施柳南区 、柳北区等城乡建设用地增减挂钩项目。

落实耕地占补制度，实现占用耕地占一补一；建立耕地保护共同责任机制，完成永久基本农田划定工作。扎实开展社会主义新农村建设，投入4.26亿元实施农村电网升级改造，稳步推进农村危房改造工作，加快乡土特色示范项目建设，扎实推进“四好”农村公路建设。

发展壮大县域经济。结合各县优势及特点，实行错位发展。柳江 、鹿寨 、柳城重点依托中心城市产业辐射，积极承接市区产业转移，发展都市农业 、特色农业和休闲农业。北部三县发展油茶 、中草药 、竹木 、林下种养等生态产业，加快发展民族特色旅游和观光休闲旅游，让“生态经济”“旅游经济”成为县域经济发展新动力。

（五）加快现代服务业发展，促进第三产业提速升级促进服务业集聚发展。启动实施服务业发展十大工程，着力打造现代物流 、汽车服务 、专业市场 、电子商务四大千亿产业集群，重点推进广西（柳钢）大宗商品交易中心 、广西汽车服务业产业园 、快递快运物流园等商贸项目建设。培育大型专业市场，加快毅德商贸城 、新柳邕农产品批发市场（二期）等市场建设。繁荣河东 、河南 、河北三大商圈，推进华润万象城 、柳南万达广场等城市综合体建设。

发展河西商圈，建成金绿洲星光大道城市广场。培育服务业企业上规模上台阶，新增限额以上企业100家。

培育发展现代服务业。大力发展科技服务 、工业研发设计 、检验检测 、中介服务等生产性服务业；加快发展家政服务 、社会养老等生活性服务业。

推进“互联网+产业”工程，重点发展“阿里巴巴·柳州”产业带，壮大电子商务业。建设柳钢工业品电商平台 、五菱汽车零部件电商平台，积极发展跨境电商，促进桂中海迅 、嘉航 、城中三大电商园区集聚发展，着力打造国家级电子商务示范基地。实施农村电商百店工程，支持京东等知名电商布局农村市场。发展会展经济，建设国际会展中心（二期），办好第六届中国-东盟（柳州）汽车 、工程机械及零部件博览会。巩固房地产市场向好态势，建立能够满足多层次需求的住房供应体系。大力发展特色餐饮业，促进柳州螺蛳粉产业标准化 、品牌化 、规模化。

积极发展现代金融业。坚持“引金入柳”，努力打造区域性现代金融中心。探索企业存量资产证券化，逐步提高市属国有资本证券化率。扩大政策性农业保险覆盖面，开展小额贷款保证保险试点。推进“政银企”合作，建立创业投资引导基金，拓宽中小企业融资渠道。培育和支持企业上市，争取3家以上企业在“新三板”挂牌。

鼓励各县设立“创业引导基金”，与金融机构共建农村综合金融服务中心。

探索设立风险补偿基金，发挥市级风险补偿资金作用，建立政府 、银行 、担保 、再担保四方风险分担机制，防范和打击非法集资，维护金融安全和社会稳定。

加快旅游名城建设。推进元宝山—贝江旅游开发，年内实现华侨城开园，争取百里柳江 、程阳八寨创建成为国家5A级景区。完善旅游信息 、旅客集散中心等公共服务体系，加强旅游行业服务品牌建设，争创国家旅游标准化示范县。坚持“引客入柳”，扩大与高铁沿线城市的旅游合作，做好境内外重点客源市场营销，全年旅游收入增长20%以上。

（六）培育发展柳州都市圈，建设生态宜居现代城市促进柳东新区优化发展。完善柳东新区规划体系，优化新区资源配置，强化产业驱动 、创新驱动 、环境驱动和教育驱动，着力打造成为产城融合的新样板。推进基础设施互联互通，建成柳东大道延长线，实施曙光大道三门江立交改扩建 、北环高速连接线工程。完善新区供水 、燃气 、污水处理等市政设施，推进教育 、医疗 、文化 、公共交通 、旅游 、体育等公共服务设施建设，儿童医院投入使用，进一步聚集新区人气。

拓展对外交通网络。推进柳州站站房扩建 、柳州东站还建工程等项目，加快焦柳铁路怀化至柳州段电气化改造，推进柳州至广州铁路（柳州段） 、柳州至韶关铁路等项目规划落地建设。

加快柳州至梧州高速公路 、柳州至南宁高速公路改扩建等项目建设。实施柳江Ⅱ级航道整治，建设官塘作业区一期工程和江口作业区一期工程，提升港口集疏运能力。完成柳州白莲机场航站楼及配套设施扩建工程项目建设。

增强城市运行保障能力。启动新一轮城市总体规划修编，继续创建国家公交都市示范城市，推进轨道交通开工建设。进一步完善防洪排涝体系，力争成为国家“海绵城市”创建试点市。实施城中村改造 、危旧房改造和老旧住宅小区 、“三无”小区综合整治。建设完善市政道路 、慢行交通系统 、绿道工程 、城市公交体系 、智能停车场 、汽车充电桩等设施。加快白沙污水处理厂（二期） 、龙泉山污水处理厂（三期）等项目建设，城市污水收集处理率提高到93%以上。

打造绿色生态优美环境。实施“花园城市”2.0版建设，启动规划建绿 、管理强绿 、文化彰绿 、科技兴绿 、依法治绿 、品牌升绿“六大绿色工程”，积极创建国家级生态园林城市。

全面推进三门江森林公园保护性建设，提升城市“绿肺”功能。实施绿色制造重点项目，推进柳北循环经济产业园“三废”综合利用 、鹿寨国家级循环化改造示范试点产业发展，加快“城市矿产”示范基地 、汽车城江口工业园建设，推动柳化 、柳州电厂退城进园升级改造，努力打造国家生态文明先行示范区。做好大气 、水 、重金属等污染防治工作，严控污染物排放，严格环保监管。深入开展“美丽柳州·生态乡村”活动，巩固“生态乡村”建设成果，持续推进“宜居乡村”“幸福乡村”建设。全面建成柳江“乡约·藕遇”、柳南“金色太阳”两个市级“美丽柳州”乡村建设综合示范区和53个市级“美丽柳州”乡村建设综合示范村屯。

提升城市管理水平。构建城市管理长效机制，行使相对集中执法，加强数字城管建设，构建“大城管”新格局。加大“两违”打击查处力度，创建无违建社区（村屯） 、无违建街道(乡镇)。开展城市扬尘治理，强化密闭化运输管理，推行使用新型智能环保渣土车。实施智慧城市建设，构建“智慧柳州”地理信息云平台，建立道路健康评价系统。加强文明城市建设，不断提升城市形象。

（七）全面深化改革，扩大开放合作，进一步增强发展活力深化各项领域改革。按照优化存量 、引导增量 、有效减量的要求，加快去产能 、去库存 、去杠杆 、降成本 、补短板，推进结构性改革，提高供给体系质量和效率。做好行政体制改革，优化完善行政审批工作机制体制，进一步厘清政府 、市场 、社会之间的关系。打好国资国企改革攻坚战，完善国有资本经营预算制度，进一步优化国资监管，努力实现市本级经营性国有资产监管工作全覆盖；完善现代企业制度，推进国有企业负责人薪酬制度改革，实施国有产权进场交易制度，加快政府投资公司整合重组，积极推进国有企业改制和上市培育发展。大力发展混合所有制经济，推动国有资本与社会资本相互参股经营。制定公布政府权责清单，让各类市场主体平等参与竞争。深入推进商事登记制度改革，降低市场主体准入门槛，依法加强市场监管。深化国税 、地税征管体制改革，进一步便民办税 、规范征管。鼓励金融机构开展林权 、农民住宅 、土地承包经营权等抵押质押贷款。

推进投融资体制改革，积极实施政府和社会资本合作（PPP）模式，支持社会资本参与重大基础设施和公共服务领域投资。

促进区域经济交流与合作。对接国家实施“一带一路”建设，探索建立沿线城市联盟。积极参与泛珠合作 、珠江—西江经济带建设，推动粤桂黔高铁经济带合作试验区（广西园）落户柳东新区，加强与佛山市全方位交流合作，主动承接产业转移，促进产业集聚和产业链延伸。开展“柳来河”基础设施合作建设，联合培育发展特色产业，推动形成产业互动互联的城市圈。

着力扩大对外贸易。优化外贸主体结构，鼓励支持中小微企业从事对外贸易，拓展发展新空间。积极申报国家出口汽车及零部件质量安全示范区，改善汽车产品出口服务环境。实施加工贸易倍增计划，发展新型贸易方式，促进外贸转型发展。实施积极进口促进战略，加快申报保税物流中心，建设电子口岸，争取开通航空口岸，促进贸易便捷通关。

（八）着力保障改善民生，不断增进人民群众福祉完善社会保障体系。落实促进就业创业政策，突出抓好高校毕业生 、农民工 、城镇困难人员等重点群体就业，新增城镇就业5万人，农村劳动力转移就业5万人。推进机关事业单位社保制度改革，加快社会保障卡发放，提升社会保障信息化建设水平。

做好社会救助精准识别，帮扶救助孤寡老人 、留守老人 、残疾人等生活困难群众，编织广覆盖 、多支撑 、更惠民 、能兜底的社会保障网。

加快民生工程建设。稳步推进各类保障性住房建设，做好棚户区改造，实施双和袜厂 、灯泡厂等职工危旧房改造项目，竣工仁和家园 、馨雅小苑等保障性住房小区。以桂北休闲旅游养生养老产业示范区建设为契机，推进养老综合改革试验区建设，促进各县建设综合福利中心，将乡镇敬老院打造成为农村养老服务中心。加大民生工作和为民办实事力度，积极谋划和落实一批便民 、利民 、惠民项目，办成一批让人民群众直接享受到实惠的实事好事。

进一步创新社会治理。建设“平安柳州”，实施“天网”工程扩容建设，将县级天网 、社会单位自建视频系统接入市级平台。开展“神剑一号”专项行动，加强消防 、交通 、危爆物品 、出租房 、物流业等安全管理。深入实施“领导干部包案”制度，做好矛盾纠纷化解工作。加强食品药品监管，创建“食品安全城市”和“农产品质量安全县”，防范食品药品安全风险。健全安全生产“党政同责 、一岗双责”工作机制，推进“打非治违”及安全专项整治行动，构建隐患排查治理长效机制，重点行业领域隐患排查上报率达100%。

（九）繁荣发展社会各项事业，共享均等化公共服务优化创新创业环境。促进“大众创业 、万众创新”，积极建设创业孵化基地 、众创空间 、农民工创业园，激发各类就业群体的创业动能。进一步完善支撑创新的人才体系，引进创新型人才 、高端人才，培育本土人才。

实施科技企业融资 、科技人才激励政策，搭建科技公共服务平台，最大限度把科技成果转化为现实生产力。

发展文化体育事业。大力发展文艺事业，继续办好“中外优秀剧目月月演”等文化惠民项目，推动3D舞蹈诗《侗寨人家》世界巡演。发展壮大文化产业，规划布局创意产业园 、绿色印刷产业园，推进东盟国际龙城府文化休旅世界等产业项目建设。引导社会力量参与公共文化建设，深入挖掘历史传统 、民族风情 、工业创意等文化资源，积极创建广西特色文化 产业示范县。开展历史文化名城保护，传承发展非物质文化，推动柳江人遗址申报成为国家文物保护单位。

实施新一轮全民健身计划，办好2016年世界水上极速运动大赛等重大体育赛事，扩大国际水上狂欢节 、芦笙斗马节 、侗族多耶节等特色节庆活动的文化效应，不断丰富人民群众精神文化生活。

优先发展教育事业。坚持立德树人，按照“学前教育促普惠 、义务教育抓均衡 、普通高中扩优势 、职业教育强品牌”的思路，加强教师队伍建设，办人民满意的教育。继续推进学前教育三年行动计划，新增一批示范性幼儿园和集团化办园试点，争取学前教育三年毛入园率达到80%。推动义务教育均衡发展，健全集团化办学学区制管理 、县属轮教等工作机制，缩小城乡教育差距。积极发展特殊教育，提高适龄残疾儿童入学率。引导普通高中特色化 、品牌化 、多元化发展，不断提高学生综合素质。加快发展高等教育，推动广西科技大学鹿山学院向应用型本科院校转型升级。构建现代职业教育体系，建设广西（柳州）职业技能公共实训基地，全面推进现代学徒制试点工作，推动学历教育向职业资格准入转变。

促进医疗卫生 、人口等事业发展。

把健康理念贯穿于城市规划之中，推进健康城市建设。促进中医药事业发展，提高医疗救助 、卫生监督 、疾病防控 、妇幼保健工作水平。加快城市公立医院改革，探索建立公立医院绩效考评 、医务人员薪酬 、医疗联合体等制度，健全分级诊疗制度，简化城镇医疗保险报销程序，引导患者有序就医。全面实施一对夫妇可生育两个孩子政策，促进人口健康均衡发展。

积极争创全国文明城市，提高市民文明素质，出台文明交通失信行为惩戒办法，营造良好文明环境。

推进经济建设与国防建设融合发展。积极支持军队改革，注重在经济建设中贯彻国防需求，深入开展国防教育，增强国防意识，扎实推进国防动员准备和后备力量建设。进一步做好征兵工作，切实加强军事设施保护。

开展“双拥”创建活动，巩固发展军政军民团结良好局面，确保实现全国双拥模范城“八连冠”。

（十）进一步改进作风，全面加强法治政府建设深入推进依法行政。积极开展地方立法工作，建立完善政府立法工作机制，提高立法工作质量和效率。严格按照法定权限和程序履行政府职能，健全市 、县 、乡三级政府法律顾问制度。全面实施“七五”普法，做好法制宣传教育。做好行政复议和行政应诉工作，切实保障公民合法权益。完善城乡基层组织自治和服务功能，激发基层组织发展活力。认真执行人大决议决定，自觉接受市人大及其常委会的法律监督和工作监督，主动接受市政协的民主监督，认真办理人大议案 、建议和政协提案。广泛听取民主党派 、工商联 、无党派人士意见，支持工会 、共青团 、妇联等人民团体发挥作用。大力推行政务公开 、政府信息公开，全面接受人民监督。

建设清正廉洁政府。深入践行“三严三实”要求，认真落实党风廉政建设“一岗双责”，严格执行廉洁从政各项规定，严肃查处“四风”和腐败问题。厉行节约，严格控制一般性支出，确保“三公”经费继续下降。加强对重大政策措施贯彻落实 、预算执行 、项目招投标 、土地出让 、政府采购等重点领域 、关键环节的行政监察和审计监督，从严查处违纪违法案件，不断提高拒腐防变和抵御风险能力。

坚持勤政务实为民。深入贯彻落实中央和自治区 、市委改进作风规定，提高政府工作效能。强化重要决策部署和重大事项推进的督查落实，严格行政问责，提高政府执行力和公信力。

严格政府工作目标考核，健全科学有效的绩效考评体系，充分发挥绩效考核的导向激励作用。强化为民服务意识，坚持开展“市长接待日”“市长企业接待日”“中小企业服务月”和领导公开大接访活动，密切与人民群众的血肉联系。

各位代表，我们正处在改革与奋进的伟大时代，全面实施“十三五”规划的新征程已经开启。让我们紧密团结在以习近平同志为总书记的党中央周围，在市委的坚强领导下，团结和依靠全市各族人民，坚定信心，主动作为，攻坚克难，为柳州在全区率先全面建成小康社会 、打造西江经济带龙头城市而努力奋斗！
